# Associations of dietary fiber intake with chronic inflammatory airway diseases and mortality in adults: a population-based study

**DOI:** 10.3389/fpubh.2023.1167167

**Published:** 2023-05-26

**Authors:** Shanhong Lin, Ning Zhu, Shengmin Zhang

**Affiliations:** ^1^Department of Ultrasound, The First Affiliated Hospital of Ningbo University, Ningbo, China; ^2^Department of Respiratory and Critical Care Medicine, The First Affiliated Hospital of Ningbo University, Ningbo, China

**Keywords:** dietary fiber, CIAD, asthma, chronic bronchitis, COPD, mortality, NHANES 4/22

## Abstract

**Objective:**

The objective of this study was to investigate the potential association between dietary fiber intakes and the prevalence of chronic inflammatory airway diseases (CIAD), as well as mortality in participants with CIAD.

**Methods:**

Data was collected from the National Health and Nutrition Examination Survey (NHANES) 2013–2018, with dietary fiber intakes being calculated as the average of two 24-h dietary reviews and divided into four groups. CIAD included self-reported asthma, chronic bronchitis, and chronic obstructive pulmonary disease (COPD). Through December 31, 2019, mortality was identified from the National Death Index. In cross-sectional studies, multiple logistic regressions were used to assess dietary fiber intakes associated with the prevalence of total and specific CIAD. Dose–response relationships were tested using restricted cubic spline regression. In prospective cohort studies, cumulative survival rates were calculated using the Kaplan–Meier method and compared using log-rank tests. Multiple COX regressions were used to assess dietary fiber intakes associated with mortality in participants with CIAD.

**Results:**

A total of 12,276 adults were included in this analysis. The participants had a mean age of 50.70 ± 17.4 years and was 47.2% male. The prevalence of CIAD, asthma, chronic bronchitis, and COPD were 20.1, 15.2, 6.3, and 4.2%, respectively. The median daily consumption of dietary fiber was 15.1 [IQR 10.5, 21.1] g. After adjusting for all confounding factors, linear and negative associations were observed between dietary fiber intakes and the prevalence of total CIAD (OR = 0.68 [0.58–0.80]), asthma (OR = 0.71 [0.60–0.85]), chronic bronchitis (OR = 0.57 [0.43–0.74]) and COPD (OR = 0.51 [0.34–0.74]). In addition, the fourth quartile of dietary fiber intake levels remained significantly associated with a decreased risk of all-cause mortality (HR = 0.47 [0.26–0.83]) compared to the first quartile.

**Conclusion:**

Dietary fiber intakes were found to be correlated with the prevalence of CIAD, and higher dietary fiber intakes were associated with a reduced mortality in participants with CIAD.

## 1. Introduction

Chronic inflammatory airway diseases (CIAD), including asthma, chronic bronchitis, and chronic obstructive pulmonary disease (COPD), have long been a major public health concern (1). With the emergence of industrialization, urbanization, and an aging population, the impact of air pollution, tobacco exposure, and unhealthy lifestyles on respiratory ailments has manifested gradually, with the incidence and mortality of many chronic respiratory diseases escalating annually. After cardiovascular diseases and cancers, chronic respiratory diseases were the third leading cause of death in 2017, accounting for 7.0% of all-cause mortality, with asthma and COPD being the most prevalent chronic respiratory diseases (1). In 2019, the global burden of disease reported that there were over 260 million poorly controlled asthma cases that resulted in 455,000 deaths (2). Additionally, COPD epidemic cases caused 3.3 million deaths out of a total of 212.3 million cases (3). Chronic airway inflammation is one of the primary pathogenesis-related processes.

As awareness and research have increased, the importance of dietary fiber in the prevention of diseases has grown. Dietary fiber, which is an important nutrient and derived primarily from grains, fruits, and vegetables, is not broken down in the small intestine by endogenous hydrolytic enzymes and remains relatively intact in structure as it enters the colon, where it can be partially or fully fermented (4). Dietary fiber is divided into soluble fiber and insoluble fiber. Moreover, dietary fiber has been shown to regulate intestinal flora and possess anti-inflammatory properties (5). Due to its own unique properties, dietary fiber plays a critical role in preserving and promoting human health. Not only does it reduce the risk of numerous digestive disorders, such as esophageal cancer (6), inflammatory bowel disease (7), and colorectal cancer (8), but it also defends against other diseases, such as hypertension (9), diabetes (10), cardiovascular disease (11), and ovarian cancer (12). Though extensive research has been conducted on the protective effects of dietary fiber on a range of diseases, the association with lung disease has received less attention, and there are no clear and uniform conclusions (13).

Dietary fiber has anti-inflammatory and antioxidant properties, and may have a positive influence on CIAD (14, 15). A study indicated that a higher intake of total dietary fiber was associated with lower asthma prevalence and fewer asthma symptoms (16), and might diminish allergic reactions in the lungs (17). Another study suggested that a diet rich in fiber-containing foods might play a role in improving lung health (18). Additionally, higher dietary fiber intakes reduced the risk of COPD in current and former smokers, but not in never-smokers (19). Although studies have shown that higher fiber intakes reduced all-cause and cause-specific mortality in different populations (20, 21), no research have reported an association between dietary fiber and mortality in patients with CIAD.

Using data from the National Health and Nutrition Examination Survey (NHANES) 2013–2018, this study examined the association between dietary fiber intake and the prevalence of CIAD (including asthma, chronic bronchitis, and COPD). In addition, we also analyzed the relationship between dietary fiber and mortality in participants with CIAD to provide scientific evidence and recommendations for the prevention and treatment of respiratory diseases.

## 2. Materials and methods

### 2.1. Study population

NHANES is a national status survey conducted by the National Center for Health Statistics to assess the health status of people in American communities over a two-year period (22). NHANES collects data from participants through two components: an interview and a health examination. The interview collects information including basic demographic characteristics, socio-economic characteristics, dietary information, and disease history through a standardized questionnaire. The health examination is conducted by professional medical staff in a Mobile Examination Centre (MEC) and focuses on collecting basic physical and biochemical examinations and other medically relevant information. NHANES is a publicly available database that allows researchers worldwide to freely access relevant survey data for research analysis. The National Center for Health Statistics (NCHS) Research Ethics Review Board approved the research protocols, and all participants gave informed consent.

In this study, we analyzed data from NHANES 2013–2018. We used participants aged >18 years with two validated 24-h dietary fiber recall data. Participants who were pregnant, lacking data for assessment of CIAD, had an extreme energy intake (men: >4,200 or < 800 kcal/day; women: >3,500 or < 500 kcal/day) (23), and had no follow-up information were excluded.

### 2.2. Assessment of dietary fiber

Through two 24-h dietary recalls, two dietitians collected dietary intake data from the participants. The first dietary recall interview was conducted in MEC. The second dietary recall information was collected by telephone interview 3–10 days later. The dietary data collected from the two 24-h dietary reviews were combined with data from the United States Department of Agriculture (USDA) Food and Nutrition Database to calculate the daily intake of food and various nutrients for the participants (24). In this study, daily dietary fiber intakes (g/day) were calculated as the average of two 24-h dietary reviews and divided into four groups according to quartiles.

### 2.3. Assessment of chronic inflammatory airway diseases

In this study, CIAD included self-reported asthma, chronic bronchitis, and COPD. On the questionnaire, participants were asked if they had been told by a doctor or other medical practitioner that you have or have had asthma, chronic bronchitis, or COPD, and those who answered “yes” were defined as participants with this disease.

### 2.4. Assessment of mortality

In prospective cohort studies, study participants who had died were identified by linkage to the National Death Index (NDI). As of December 31st, 2019, we obtained all-cause mortality records for participants via the 2019 Linked Mortality File (LMF), which reports the latest associations made between selected NCHS surveys and the NDI.

### 2.5. Covariates

Information regarding participants’ baseline data was collected through questionnaires and laboratory tests, including age (18–39, 40–59, or ≥ 60 years), gender (male or female), educational attainment (below high school, high school, or above high school), race/ethnicity (Mexican American, other Hispanic, non-Hispanic white, non-Hispanic black, or other race), and body mass index (<25.0, 25.0–29.9, or > 29.9 kg/m^2^). Income was measured using the poverty-income ratio (PIR; the ratio of family income divided by a poverty threshold specific for family size using guidelines from the US Department of Health and Human Services) and categorized as ≤1.0, 1.1–3.0, and > 3.0 (25). Never smokers were identified as those who reported smoking <100 cigarettes over the course of their lifetime. Those who smoked >100 cigarettes in their lifetime were classified as current smokers, and those who smoked >100 cigarettes and had quit smoking were labeled as former smokers (26). Drinking status was classified as nondrinker, low-to-moderate drinker (<2 drinks/day in men and < 1 drink/day in women), or heavy drinker (≥2 drinks/day in men and ≥ 1 drinks/day in women) (26). Physical activity was divided into three groups: inactive (no leisure-time physical activity), insufficiently active [moderate activity 1–5 times per week with metabolic equivalents (MET) 3–6 or vigorous activity 1–3 times per week with MET >6], and active (those who had more moderate or vigorous activity than the previously mentioned) (26, 27).

### 2.6. Statistical analysis

The categorical variables were represented numerically (percentages). In order to normalize their distributions, the dietary fiber intake levels were log-transformed. Missing values for covariates were filled using the imputation approach, which was based on a “mice” package of the Random Forest algorithm. We performed all statistical analyses using R software (version 4.2.0).

In cross-sectional studies, multiple logistic regression model was employed to ascertain the adjusted odds ratios (ORs) and 95% confidence intervals (CIs) of the association between quartiles of dietary fiber intake levels and the prevalence of total and specific CIAD. To further explore the dose–response curves of this association, restricted cubic spline regression analysis was conducted with knots set at the 10th, 50th, and 90th percentiles of dietary fiber intake levels. Additionally, stratified analyses were conducted across numerous subgroups. In prospective cohort studies, using the Kaplan–Meier method, cumulative survival rates were calculated and log-rank tests were utilized to compare the survival rates between four groups of participants, which were divided based on the quartiles of dietary fiber intake levels. Additionally, multiple COX regressions were implemented to calculate adjusted hazard ratios (HRs) and 95% CIs in relation to mortality of participants with CIAD.

## 3. Results

### 3.1. Characteristics of study participants

**Figure 1:**
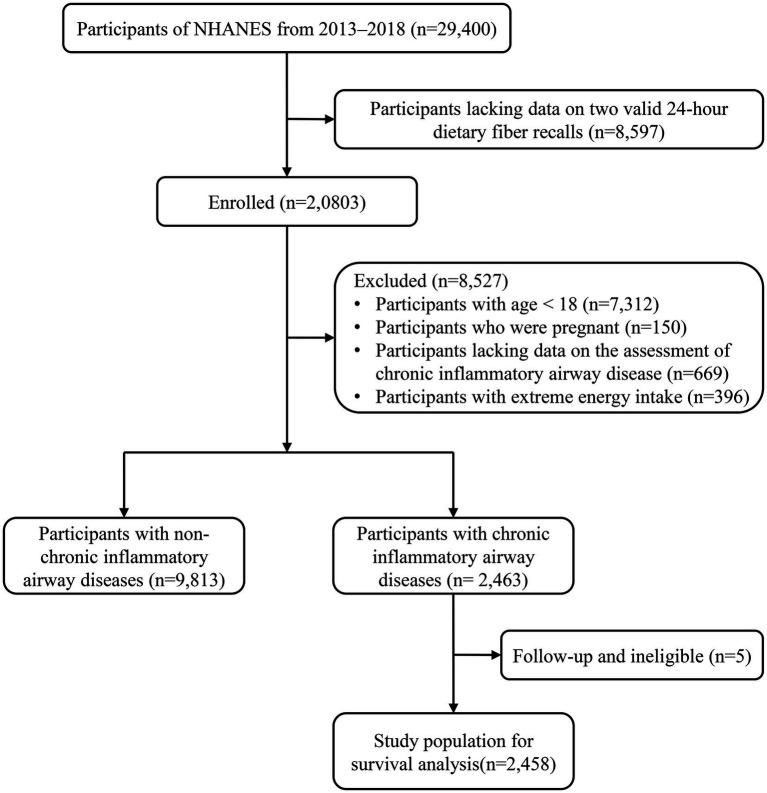
Flowchart of the study participants.

Between 2013 and 2018, 29,400 participants participated in the NHANES, of which 8,597 lacked data on two valid 24-h dietary fiber recalls. Subsequently, we removed participants younger than 18 (*n* = 7,312), those who were pregnant (*n* = 150), those who lacked data on CIAD assessment (*n* = 669), and those who had excessive energy consumption (*n* = 396), leaving 12,276 eligible participants, of whom 9,813 had non-CIAD and 2,463 participants with CIAD. In addition, the five participants with CIAD whom we failed to interview were excluded, resulting in a total of 2,458 participants with CIAD for survival analysis (Figure 1).

Table 1 displays the baseline characteristics of the quartiles of dietary fiber intake levels for adult participants in the NHANES 2013–2018. The study population had a mean age of 50.70 ± 17.4 years, was 47.2% male, and was mainly non-Hispanic white (39.3%). The prevalence of CIAD, asthma, chronic bronchitis, and COPD were 20.1, 15.2, 6.3, and 4.2%, respectively. The median daily consumption of dietary fiber was 15.1 [IQR 10.5, 21.1] g. Compared to the 3,060 participants in the first quartile of dietary fiber levels, the 3,072 participants in the fourth quartile were more likely to be young to middle-aged males, to have higher education and income levels, to be low-to-moderate drinkers, non-smokers, physically active, to have a higher energy intake, and to have a lower prevalence of CIAD, asthma, chronic bronchitis, and COPD.

**Table 1:** Characteristics of adult participants with available data on dietary fiber intakes in NHANES 2013–2018.

Characteristics	Total	Quartiles of dietary fiber intake levels, g/day				*p-*value
		<10.5	10.5–15.0	15.1–21.1	>21.1	
Participants	12,276	3,060	3,076	3,068	3,072	
Age, years						<0.001
18–39	3,753 (30.6)	998 (32.6)	995 (32.3)	884 (28.8)	876 (28.5)	
40–59	4,132 (33.7)	1,038 (33.9)	946 (30.8)	1,043 (34.0)	1,105 (36.0)	
≥60	4,391 (35.8)	1,024 (33.5)	1,135 (36.9)	1,141 (37.2)	1,091 (35.5)	
Male, %	5,792 (47.2)	1,160 (37.9)	1,353 (44.0)	1,474 (48.0)	1805 (58.8)	<0.001
Race/ethnicity, %						<0.001
Mexican American	1713 (14.0)	246 (8.0)	323 (10.5)	450 (14.7)	694 (22.6)	
Other Hispanic	1,262 (10.3)	299 (9.8)	309 (10.0)	311 (10.1)	343 (11.2)	
Non-Hispanic White	4,825 (39.3)	1,225 (40.0)	1,280 (41.6)	1,276 (41.6)	1,044 (34.0)	
Non-Hispanic Black	2,683 (21.9)	935 (30.6)	761 (24.7)	590 (19.2)	397 (12.9)	
Other race	1793 (14.6)	355 (11.6)	403 (13.1)	441 (14.4)	594 (19.3)	
Education level, %						<0.001
Below high school	2,309 (18.8)	663 (21.7)	523 (17.0)	520 (16.9)	603 (19.6)	
High school	2,816 (22.9)	865 (28.3)	776 (25.2)	668 (21.8)	507 (16.5)	
Above high school	7,151 (58.3)	1,532 (50.1)	1777 (57.8)	1880 (61.3)	1962 (63.9)	
Family income-to-poverty ratio, %						<0.001
≤1.0	2,459 (20.7)	832 (28.3)	633 (21.2)	495 (16.7)	499 (16.7)	
1.1–3.0	4,801 (40.4)	1,270 (43.2)	1,237 (41.4)	1,221 (41.1)	1,073 (36.0)	
>3.0	4,619 (38.9)	835 (28.4)	1,121 (37.5)	1,254 (42.2)	1,409 (47.3)	
Smoking status, %						<0.001
Never smoker	7,057 (57.5)	1,513 (49.4)	1748 (56.8)	1842 (60.0)	1954 (63.6)	
Former smoker	2,997 (24.4)	669 (21.9)	740 (24.1)	786 (25.6)	802 (26.1)	
Current smoker	2,222 (18.1)	878 (28.7)	588 (19.1)	440 (14.3)	316 (10.3)	
Drinking status, %						<0.001
Nondrinker	3,049 (24.8)	768 (25.1)	745 (24.2)	767 (25.0)	769 (25.0)	
Low-to-moderate drinker	8,352 (68.0)	2000 (65.4)	2,100 (68.3)	2,110 (68.8)	2,142 (69.7)	
Heavy drinker	875 (7.1)	292 (9.5)	231 (7.5)	191 (6.2)	161 (5.2)	
Body mass index, kg/m^2^						<0.001
<25.0	3,270 (26.6)	755 (24.7)	798 (25.9)	802 (26.1)	915 (29.8)	
25.0–29.9	3,920 (31.9)	913 (29.8)	887 (28.8)	1,035 (33.7)	1,085 (35.3)	
>29.9	5,086 (41.4)	1,392 (45.5)	1,391 (45.2)	1,231 (40.1)	1,072 (34.9)	
Physical activity, %						<0.001
Inactive	3,150 (25.7)	960 (31.4)	862 (28.0)	728 (23.7)	600 (19.5)	
Insufficiently active	3,985 (32.5)	925 (30.2)	1,022 (33.2)	1,041 (33.9)	997 (32.5)	
Active	5,141 (41.9)	1,175 (38.4)	1,192 (38.8)	1,299 (42.3)	1,475 (48.0)	
Energy intake, kcal/day	1884.5 [1454.9, 2411.0]	1418.3 [1103.5, 1807.1]	1781.3 [1438.0, 2194.0]	2025.8 [1644.5, 2484.9]	2377.3 [1928.0, 2888.0]	<0.001
Chronic inflammatory airway diseases, %	2,463 (20.1)	793 (25.9)	634 (20.6)	565 (18.4)	471 (15.3)	<0.001
Asthma, %	1863 (15.2)	590 (19.3)	467 (15.2)	429 (14.0)	377 (12.3)	<0.001
Chronic bronchitis, %	775 (6.3)	295 (9.6)	203 (6.6)	158 (5.1)	119 (3.9)	<0.001
Chronic obstructive pulmonary disease, %	515 (4.2)	197 (6.4)	150 (4.9)	103 (3.4)	65 (2.1)	<0.001

Continuous variables without a normal distribution are presented as medians (interquartile ranges). Categorical variables are presented as numbers (percentages).

### 3.2. Associations between dietary fiber intakes and the prevalence of CIAD

**Figure 2:**
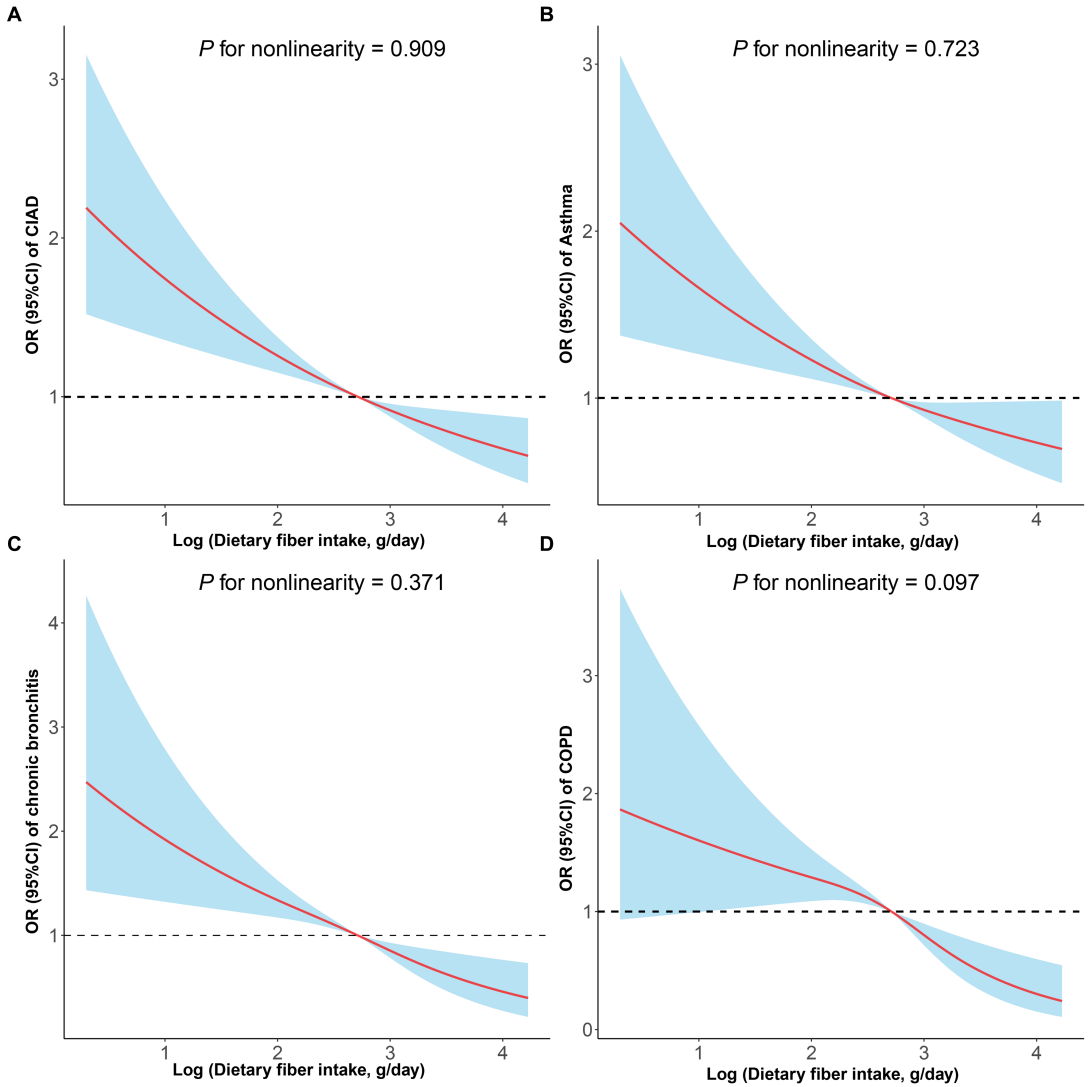
Restricted cubic spline analyses the association of dietary fiber intake levels and the prevalence of total and specific chronic inflammatory airway diseases (CIAD) **(A)**, including asthma **(B)**, chronic bronchitis **(C)**, and COPD **(D)**. Adjusted for age (18–39, 40–59, or ≥ 60), sex (male or female), and race (Mexican American, Other Hispanic, Non-Hispanic White, Non-Hispanic Black, or Other), education level (below high school, high school, or above high school), family income-to-poverty ratio (≤1.0, 1.1–3.0, or > 3.0), smoking status (never smoker, former smoker, or current smoker), drinking status (nondrinker, low-to-moderate drinker, or heavy drinker), BMI (<25.0, 25.0–29.9, or > 29.9), energy intake levels (in quartiles), and physical activity (inactive, insufficiently active, or active).

In cross-sectional studies, dietary fiber intake levels were classified into quartiles, with the lowest quartile being the reference category, and assessed for their association with the prevalence of total and specific CIAD (Table 2). The crude model revealed a negative correlation between dietary fiber intake quartiles and the prevalence of both total and specific CIAD. After adjusting for age, sex, and race, this relationship remained statistically significant. Compared to the reference quartile, the dietary fiber intake in the second, third and fourth quartiles in Model 2 was associated with the prevalence of total CIAD, asthma and COPD. The association between dietary fiber intake and COPD was attenuated, but dietary fiber intake in the third and fourth quartiles was still associated with the prevalence of COPD. After correcting for all confounding factors, we found that dietary fiber intake levels in the fourth quartile were significantly associated with a lower prevalence of total CIAD (OR = 0.68 [0.58–0.80]), asthma (OR = 0.71 [0.60–0.85]), chronic bronchitis (OR = 0.57 [0.43–0.74]) and COPD (OR = 0.51 [0.34–0.74]) compared to the lowest quartile. In addition, the linear and negative associations were observed between dietary fiber intake levels and the prevalence of CIAD (P for nonlinearity = 0.909, Figure 2A), asthma (P for nonlinearity = 0.723, Figure 2B), bronchitis (P for nonlinearity = 0.371, Figure 2C) and COPD (P for nonlinearity = 0.097, Figure 2D). Stratified analysis showed no significant interaction between dietary fiber intakes and any strata variables (age, sex, race, education level, family PIR, smoking status, BMI, and physical activity) with the prevalence of CIAD (Table 3). In addition, we found a negative correlation between dietary carotenoid intake levels and inflammatory markers (including, CRP, leukocyte count, neutrophil count, Table 4).

**Table 2:** ORs (95% CIs) of the prevalence of chronic inflammatory airway diseases (CIAD) according to quartiles of dietary fiber intake levels among adults in NHANES 2013–2018 (*n* = 12,276).

	Dietary fiber intake levels, g/day				
	Quartile 1	Quartile 2	Quartile 3	Quartile 4	*P*_trend_
CIAD					
Crude	1 [Reference]	0.75 (0.67–0.85)	0.65 (0.57–0.73)	0.52 (0.46–0.59)	<0.001
Model 1	1 [Reference]	0.76 (0.68–0.86)	0.68 (0.60–0.77)	0.60 (0.52–0.68)	<0.001
Model 2	1 [Reference]	0.81 (0.71–0.92)	0.76 (0.66–0.87)	0.68 (0.58–0.80)	<0.001
Asthma					
Crude	1 [Reference]	0.76 (0.66–0.87)	0.69 (0.60–0.79)	0.59 (0.51–0.68)	<0.001
Model 1	1 [Reference]	0.79 (0.69–0.90)	0.74 (0.65–0.85)	0.69 (0.60–0.80)	<0.001
Model 2	1 [Reference]	0.80 (0.69–0.93)	0.77 (0.66–0.90)	0.71 (0.60–0.85)	<0.001
Chronic bronchitis					
Crude	1 [Reference]	0.65 (0.54–0.79)	0.50 (0.41–0.61)	0.38 (0.30–0.47)	<0.001
Model 1	1 [Reference]	0.66 (0.54–0.79)	0.51 (0.41–0.62)	0.43 (0.34–0.54)	<0.001
Model 2	1 [Reference]	0.76 (0.62–0.93)	0.63 (0.50–0.80)	0.57 (0.43–0.74)	<0.001
COPD					
Crude	1 [Reference]	0.75 (0.60–0.93)	0.50 (0.39–0.63)	0.32 (0.24–0.42)	<0.001
Model 1	1 [Reference]	0.68 (0.54–0.84)	0.43 (0.33–0.55)	0.29 (0.21–0.39)	<0.001
Model 2	1 [Reference]	0.89 (0.68–1.17)	0.63 (0.46–0.87)	0.51 (0.34–0.74)	<0.001

Model 1 was adjusted for age (18–39, 40–59, or ≥ 60), sex (male or female), and race (Mexican American, Other Hispanic, Non-Hispanic White, Non-Hispanic Black, or Other).

Model 2 was adjusted for Model 1 plus education level (below high school, high school, or above high school), family income-to-poverty ratio (≤1.0, 1.1–3.0, or > 3.0), smoking status (never smoker, former smoker, or current smoker), drinking status (nondrinker, low-to-moderate drinker, or heavy drinker), BMI (<25.0, 25.0–29.9, or > 29.9), energy intake levels (in quartiles), physical activity (inactive, insufficiently active, or active).

**Table 3:** Stratified analyses of the associations between quartiles of dietary fiber intake levels and the prevalence of chronic inflammatory airway diseases (CIAD) in NHANES 2013–2018.

Subgroups	N	Dietary fiber intake levels, g/day				*P*_interaction_
		Quartile 1	Quartile 2	Quartile 3	Quartile 4	
Age, years						
18–39	3,753	1 [Reference]	0.75 (0.59–0.95)	0.78 (0.60–1.01)	0.62 (0.46–0.83)	0.704
40–59	4,132	1 [Reference]	0.80 (0.63–1.01)	0.70 (0.55–0.91)	0.72 (0.54–0.95)	
≥60	4,391	1 [Reference]	0.84 (0.68–1.04)	0.77 (0.61–0.97)	0.68 (0.53–0.89)	
Sex, %						
Male	5,792	1 [Reference]	0.73 (0.59–0.90)	0.71 (0.57–0.88)	0.64 (0.51–0.81)	0.767
Female	6,484	1 [Reference]	0.85 (0.72–1.01)	0.78 (0.65–0.94)	0.69 (0.55–0.86)	
Race, %						
Non-Hispanic White	4,825	1 [Reference]	0.87 (0.71–1.05)	0.78 (0.63–0.96)	0.83 (0.65–1.05)	0.075
Other	7,451	1 [Reference]	0.74 (0.62–0.88)	0.71 (0.59–0.86)	0.53 (0.43–0.65)	
Education level, %						
Below high school	2,309	1 [Reference]	0.82 (0.61–1.10)	0.55 (0.39–0.78)	0.58 (0.40–0.86)	0.125
High school	2,816	1 [Reference]	0.80 (0.62–1.04)	0.84 (0.63–1.11)	0.70 (0.50–0.99)	
Above high school	7,151	1 [Reference]	0.81 (0.68–0.97)	0.79 (0.66–0.96)	0.70 (0.57–0.86)	
Family PIR, %						
≤1.0	2,459	1 [Reference]	0.84 (0.65–1.09)	0.75 (0.56–1.02)	0.66 (0.47–0.93)	0.710
1.1–3.0	4,801	1 [Reference]	0.81 (0.66–0.99)	0.72 (0.58–0.90)	0.60 (0.46–0.77)	
>3.0	4,619	1 [Reference]	0.77 (0.60–0.97)	0.79 (0.62–1.01)	0.74 (0.56–0.96)	
Smoking status, %						
Never smoker	7,057	1 [Reference]	0.89 (0.73–1.07)	0.87 (0.71–1.07)	0.77 (0.62–0.97)	0.257
Former smoker	2,997	1 [Reference]	0.74 (0.58–0.96)	0.59 (0.45–0.78)	0.52 (0.38–0.70)	
Current smoker	2,222	1 [Reference]	0.79 (0.61–1.02)	0.80 (0.59–1.08)	0.76 (0.53–1.10)	
Body mass index, kg/m^2^						
<25.0	3,270	1 [Reference]	0.81 (0.61–1.06)	0.67 (0.50–0.90)	0.57 (0.41–0.80)	0.840
25.0–29.9	3,920	1 [Reference]	0.73 (0.57–0.95)	0.79 (0.61–1.02)	0.65 (0.49–0.87)	
>29.9	5,086	1 [Reference]	0.83 (0.69–0.99)	0.77 (0.62–0.94)	0.73 (0.58–0.93)	
Physical activity, %						
Inactive	3,150	1 [Reference]	1.02 (0.80–1.29)	0.9 (0.69–1.18)	0.76 (0.55–1.06)	0.396
Insufficiently active	3,985	1 [Reference]	0.72 (0.57–0.91)	0.65 (0.50–0.83)	0.63 (0.47–0.83)	
Active	5,141	1 [Reference]	0.72 (0.58–0.89)	0.73 (0.58–0.91)	0.63 (0.50–0.81)	

Analyses were adjusted for covariates age (18–39, 40–59, or ≥ 60), sex (male or female), and race (Mexican American, Other Hispanic, Non-Hispanic White, Non-Hispanic Black, or Other), education level (below high school, high school, or above high school), family income-to-poverty ratio (≤1.0, 1.1–3.0, or > 3.0), smoking status (never smoker, former smoker, or current smoker), drinking status (nondrinker, low-to-moderate drinker, or heavy drinker), BMI (<25.0, 25.0–29.9, or > 29.9), energy intake levels (in quartiles), and physical activity (inactive, insufficiently active, or active) when they were not the strata variables.

**Table 4:** Multiple linear regression associations of dietary fiber intake levels with inflammatory markers in adults.

Outcomes	β (95%CI)	*P-*value
C-reactive protein	−0.212 (−0.268, −0.156)	<0.001
White blood cell count	−0.022 (−0.033, −0.010)	<0.001
Neutrophil count	−0.029 (−0.044, −0.014)	<0.001
Lymphocyte count	0.011 (−0.003, 0.024)	0.127

Continuous dietary fiber intake levels and inflammatory markers were log-transformed.

Model was adjusted as age (18–39, 40–59, or ≥ 60), sex (male or female), race (Mexican American, Other Hispanic, Non-Hispanic White, Non-Hispanic Black or Other), education level (below high school, high school, or above high school), family income-to-poverty ratio (≤1.0, 1.1–3.0, or > 3.0), smoking status (never smoker, former smoker, or current smoker), drinking status (nondrinker, low-to-moderate drinker, or heavy drinker), BMI (<25.0, 25.0–29.9, or > 29.9), energy intake levels (in quartiles), physical activity (inactive, insufficiently active, or active).

### 3.3. Associations between dietary fiber intakes and mortality among adults with CIAD

**Figure 3:**
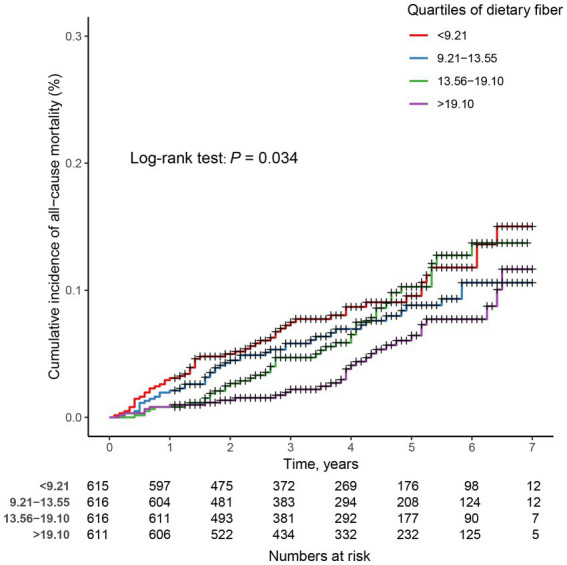
Kaplan–Meier survival curves for all-cause mortality in adults with chronic inflammatory airway diseases (CIAD) grouped by quartiles of dietary fiber intake levels. Quartiles of dietary fiber intake levels were < 9.21, 9.21–13.55, 13.56–19.10, and > 19.10 g/day, respectively.

**Figure 4:**
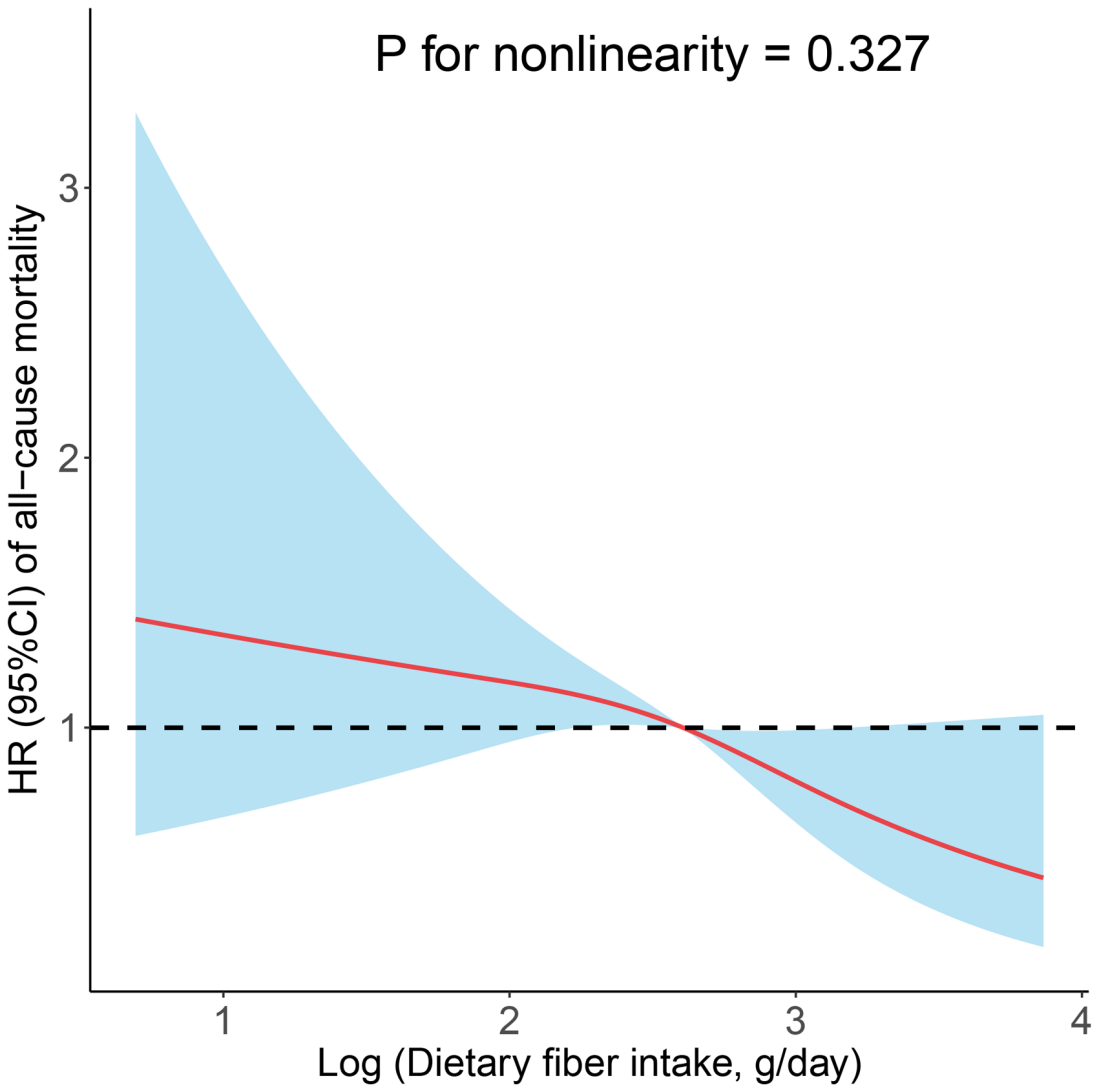
Restricted cubic spline analyses the association of dietary fiber intake levels and the risk of all-cause mortality. Adjusted for age (18–39, 40–59, or ≥ 60), sex (male or female), and race (Mexican American, Other Hispanic, Non-Hispanic White, Non-Hispanic Black, or Other), education level (below high school, high school, or above high school), family income-to-poverty ratio (≤1.0, 1.1–3.0, or > 3.0), smoking status (never smoker, former smoker, or current smoker), drinking status (nondrinker, low-to-moderate drinker, or heavy drinker), BMI (<25.0, 25.0–29.9, or > 29.9), energy intake levels (in quartiles), and physical activity (inactive, insufficiently active, or active).

In prospective cohort studies, over a median follow-up of 3.83 years, there were 175 (7.10%) all-cause deaths among 2,458 adults with CIAD. Kaplan–Meier showed that across quartiles of dietary fiber intake levels, participants in the highest quartile had the lowest risk of all-cause death (log-rank test *p* = 0.034; Figure 3). After multivariate adjustment, the fourth quartile remained significantly associated with an increased risk of all-cause mortality (HR = 0.47, 95% CI 0.26–0.83) compared with the first quartile of dietary fiber intake levels (Table 5). In addition, the linear associations were observed between dietary fiber intake levels and the risk of all-cause mortality (*P* for nonlinearity = 0.327, Figure 4).

**Table 5:** HRs (95% CIs) of all-cause mortality according to quartiles of dietary fiber intake levels among adults with chronic inflammatory airway diseases (CIAD) in NHANES 2013–2018 (*n* = 2,458).

	Quartiles of dietary fiber intake levels, g/day				
	<9.21	9.21–13.55	13.56–19.10	>19.10	*P*_trend_
No. deaths/total	54/615	44/616	45/616	32/611	
Crude	1 [Reference]	0.76 (0.51–1.14)	0.82 (0.55–1.21)	0.52 (0.34–0.81)	0.008
Model 1	1 [Reference]	0.70 (0.47–1.04)	0.73 (0.49–1.09)	0.46 (0.29–0.72)	0.001
Model 2	1 [Reference]	0.63 (0.41–0.97)	0.70 (0.44–1.12)	0.47 (0.26–0.83)	0.020

Model 1 was adjusted for age (18–39, 40–59, or ≥ 60), sex (male or female), and race (Mexican American, Other Hispanic, Non-Hispanic White, Non-Hispanic Black or Other).

Model 2 was adjusted for Model 1 plus education level (below high school, high school, or above high school), family income-to-poverty ratio (≤1.0, 1.1–3.0, or > 3.0), smoking status (never smoker, former smoker, or current smoker), drinking status (nondrinker, low-to-moderate drinker, or heavy drinker), BMI (<25.0, 25.0–29.9, or > 29.9), energy intake levels (in quartiles), physical activity (inactive, insufficiently active, or active).

## 4. Discussion

Using data from the NHANES 2013–2018, we investigated the relationship between dietary fiber intakes and the prevalence of total and specific CIAD (asthma, chronic bronchitis, and COPD) as well as all-cause mortality in adults with CIAD. Results indicated that participants in the fourth quartile of dietary fiber intakes were significantly associated with a lower prevalence of total and specific CIAD (including asthma, chronic bronchitis, and COPD) after adjustments for all confounders. Moreover, greater dietary fiber intakes reduced all-cause mortality in individuals with CIAD.

Research has demonstrated that low dietary fiber consumption is associated with decreased lung function, and a diet with high dietary fiber content may improve lung health (28). This is in line with the findings of Andrianasolo et al. (29), who observed that both soluble and insoluble dietary fiber, either derived from fruits or vegetables, were negatively linked to asthma symptom scores. Additionally, their results showed that the odds ratio (OR) for the highest quartile of total dietary fiber compared to the lowest quartile was 0.73 (95% CI 0.67–0.79) in women and 0.63 (95% CI 0.55–0.73) in men. Furthermore, numerous studies indicate that a higher intake of dietary fiber can reduce the risk of COPD (30, 31). Kaluza et al. summarized the outcomes of cohort studies on the association between dietary fiber intake and the risk of COPD in men and women, respectively (19, 32). They concluded that long-term high dietary fiber intake lowered the risk of COPD in both genders, which is consistent with our results. However, in the study by Kaluza, the protective effect of increased high-dietary fiber intake on the risk of COPD in never smokers did not achieve statistical significance, and high-dose dietary fiber intake notably reduced the risk of COPD in former and current smokers. Our stratified analysis did not reveal the above associations, likely due to the limited number of COPD cases included in the study, leading to inconclusive results.

Globally, one of the dietary risk factors for death is inadequate intake of whole grains, fruits, and vegetables (33). Whole grains, fresh fruits, and vegetables are the main sources of dietary fiber (34). According to the World Health Organization, the global average daily intake of dietary fiber is about 17 g, which is below the recommended 25 g/day. In this study, 1867 (60.8%) of individuals in Q4 meet this recommendation. Our findings suggest that a significant proportion of individuals may not be meeting this recommendation, which highlights the need for interventions to promote healthy dietary habits and increase fiber intake. Recent evidence also implies a potential inverse relationship between total dietary fiber intake and all-cause or cause-specific mortality (35–38). These investigations focused on analyzing the association between dietary fiber intake and the risk of all-cause, cardiovascular disease, diabetes, and cancer mortality in the general population. Nonetheless, no studies have investigated the effect of dietary fiber intake on the risk of all-cause mortality in patients with CIAD. In our study, those in the highest quartile of dietary fiber intake levels had the lowest risk of all-cause death, and the fourth quartile remained significantly related to a decreased risk of all-cause mortality compared with the first quartile of dietary fiber intake levels. This suggests that a suitable increment in dietary fiber intake may reduce the risk of all-cause mortality in individuals with CIAD.

Studies have demonstrated that the microbial metabolites derived from dietary fiber, such as anti-inflammatory short-chain fatty acids (SCFAs), can lessen the inflammatory effect in the lungs (39). In the initial investigation by Halnes et al. on the acute responses of soluble fiber on airway inflammation in asthma, alterations in expressions of the free fatty acid receptor gene were observed (40). The results revealed that, in comparison with the placebo group, a single intake of soluble fiber was efficacious in bringing down airway inflammation, which corresponded with an increase in SCFAs concentrations in blood and an upregulation of GRP41 and GPR43 expression. In a subsequent study of allergic airway disease, Trompette et al. discovered that a fiber-rich diet modified the composition of the intestinal microbiota by elevating the proportion of Bacteroidetes and Bifidobacteria and influenced the production of SCFAs (41). Additionally, dietary fiber is thought to enhance the gut barrier, acting as a physical and chemical barrier, thus reducing the release of inflammatory mediators into the bloodstream that can affect the lungs (42). Furthermore, certain dietary fiber, such as those found in oats, barley, and psyllium, may directly reduce inflammation in the lungs. This is thought to occur by reducing the production of pro-inflammatory molecules and increasing the production of anti-inflammatory molecules (43). Finally, dietary fiber can help to reduce oxidative stress and provide protection against air pollution, which may in turn help to reduce the risk of chronic inflammatory airway disease (44, 45).

The major advantage of this study is its large, representative sample, which enabled us to discover the association between dietary fiber intakes and the prevalence of total and specific CIAD and all-cause mortality in adults. Second, our study adjusted for the primary factors affecting lung function during the analysis, including age, smoking, BMI, physical activity, and other confounding factors. After adjusting for all confounders, our findings remained relatively stable. Third, our study used the RCS model to assess the dose–response association between dietary fiber intakes and CIAD, and a linear association was found among them. Finally, in contrast to previous studies, we analyzed the association between dietary fiber intakes and the risk of all-cause mortality in participants with CIAD.

This study has several limitations. First, this study is essentially an observational study and hence cannot establish causality. Second, dietary data collected by the 24-h dietary recall method may be subject to recall bias. There were no uniform diagnostic criteria for CIAD and no detailed disease information in the included studies. Moreover, the data were collected from the self-reported questionnaires, which are more prone to biases and should also be interpreted with caution. Third, the results of studies on the association between dietary fiber intake and the prevalence of three lung diseases were adjusted for age, sex, smoking status, and other confounding factors, but there may be unknown confounding factors affecting the results of the analyses. Fourth, although we considered the effect of smoke exposure on respiratory disease, we did not specifically quantify the amount of smoke exposure, such as how many packs of cigarettes smoked per year or serum cotinine concentrations, but simply reflected smoke exposure by classifying smokers (never smoker, former smoker, or current smoker). Finally, dietary fiber is divided into soluble fiber and insoluble fiber, and the two types of fiber may have different effects on the risk of disease. Due to the limited availability of data, we were unable to analyze them separately.

## 5. Conclusion

Our findings suggested that higher dietary fiber intakes were associated with a lower prevalence of CAID (including asthma, chronic bronchitis, and COPD). Among participants with CAID, higher dietary fiber intakes were associated with a reduced risk of all-cause mortality.

## Data availability statement

Publicly available datasets were analyzed in this study. This data can be found here: https://www.cdc.gov/nchs/nhanes/search/default.aspx.

## Ethics statement

Detailed methods and protocols for the NHANES study were approved by the CDC/NCHS Research Ethics Review Board. They are publicly available through the CDC.gov website; this includes informed consent procedures for all participants. All methods in this study were performed according to the relevant guidelines and regulations. This study was exempt from human subject ethical review as the data are freely available in the public domain.

## Author contributions

SL and NZ conceived and designed the study, extracted the data, and analyzed and interpreted the data. SL, NZ, and SZ contributed to drafting and editing the manuscript and full access to all the data in the study and took responsibility for the integrity of the data and the accuracy of the data analysis. All authors have given the final approval of the manuscript.

## Conflict of interest

The authors declare that the research was conducted in the absence of any commercial or financial relationships that could be construed as a potential conflict of interest.

## Publisher’s note

All claims expressed in this article are solely those of the authors and do not necessarily represent those of their affiliated organizations, or those of the publisher, the editors and the reviewers. Any product that may be evaluated in this article, or claim that may be made by its manufacturer, is not guaranteed or endorsed by the publisher.

## References

[1] Soriano JB Kendrick PJ Paulson KR Gupta V Abrams EM Adedoyin RA. Prevalence and attributable health burden of chronic respiratory diseases, 1990-2017: a systematic analysis for the global burden of disease study 2017. Lancet Respir Med. (2020) 8:585–96. doi: 10.1016/S2213-2600(20)30105-3, PMID: 32526187 PMC7284317

[2] Vos T Lim SS Abbafati C Abbas KM Abbasi M Abbasifard M. Global burden of 369 diseases and injuries in 204 countries and territories, 1990-2019: a systematic analysis for the global burden of disease study 2019. Lancet. (2020) 396:1204–22. doi: 10.1016/S0140-6736(20)30925-9, PMID: 33069326 PMC7567026

[3] Safiri S Carson-Chahhoud K Noori M Nejadghaderi SA Sullman MJM Ahmadian Heris J. Burden of chronic obstructive pulmonary disease and its attributable risk factors in 204 countries and territories, 1990-2019: results from the global burden of disease study 2019. BMJ. (2022) 378:e069679. doi: 10.1136/bmj-2021-069679 35896191 PMC9326843

[4] Howlett JF Betteridge VA Champ M Craig SA Meheust A Jones JM. The definition of dietary fiber - discussions at the ninth Vahouny Fiber Symposium: building scientific agreement. Food Nutr Res. (2010) 54:5750. doi: 10.3402/fnr.v54i0.5750 PMC2972185 21052531

[5] Cronin P Joyce SA O’Toole PW O’Connor EM. Dietary fibre modulates the gut microbiota. Nutrients. (2021) 13:1655. doi: 10.3390/nu13051655 34068353 PMC8153313

[6] Sun L Zhang Z Xu J Xu G Liu X. Dietary fiber intake reduces risk for Barrett’s esophagus and esophageal cancer. Crit Rev Food Sci Nutr. (2017) 57:2749–57. doi: 10.1080/10408398.2015.1067596, PMID: 26462851

[7] Liu X Wu Y Li F Zhang D. Dietary fiber intake reduces risk of inflammatory bowel disease: result from a meta-analysis. Nutr Res. (2015) 35:753–8. doi: 10.1016/j.nutres.2015.05.021, PMID: 26126709

[8] Aune D Chan DS Lau R Vieira R Greenwood DC Kampman E. Dietary fibre, whole grains, and risk of colorectal cancer: systematic review and dose-response meta-analysis of prospective studies. BMJ. (2011) 343:d6617. doi: 10.1136/bmj.d6617, PMID: 22074852 PMC3213242

[9] Whelton SP Hyre AD Pedersen B Yi Y Whelton PK He J. Effect of dietary fiber intake on blood pressure: a meta-analysis of randomized, controlled clinical trials. J Hypertens. (2005) 23:475–81. doi: 10.1097/01.hjh.0000160199.51158.cf, PMID: 15716684

[10] Kuijsten A Aune D Schulze MB Norat T van Woudenbergh GJ Beulens JW. Dietary fibre and incidence of type 2 diabetes in eight European countries: the EPIC-inter act study and a meta-analysis of prospective studies. Diabetologia. (2015) 58:1394–408. doi: 10.1007/s00125-015-3585-9, PMID: 26021487 PMC4472947

[11] Threapleton DE Greenwood DC Evans CE Cleghorn CL Nykjaer C Woodhead C. Dietary fibre intake and risk of cardiovascular disease: systematic review and meta-analysis. BMJ. (2013) 347:f6879. doi: 10.1136/bmj.f6879, PMID: 24355537 PMC3898422

[12] Xu H Ding Y Xin X Wang W Zhang D. Dietary fiber intake is associated with a reduced risk of ovarian cancer: a dose-response meta-analysis. Nutr Res. (2018) 57:1–11. doi: 10.1016/j.nutres.2018.04.011, PMID: 30122191

[13] Young RP Hopkins RJ. Is the “Western diet” a new smoking gun for chronic obstructive pulmonary disease? Ann Am Thorac Soc. (2018) 15:662–3. doi: 10.1513/AnnalsATS.201802-131ED, PMID: 29856249

[14] Venter C Meyer RW Greenhawt M Pali-Schöll I Nwaru B Roduit C. Role of dietary fiber in promoting immune health-an EAACI position paper. Allergy. (2022) 77:3185–98. doi: 10.1111/all.15430, PMID: 35801383

[15] Tan JK Macia L Mackay CR. Dietary fiber and SCFAs in the regulation of mucosal immunity. J Allergy Clin Immunol. (2022) 151:361–70. doi: 10.1016/j.jaci.2022.11.007 36543697

[16] Saeed MA Gribben KC Alam M Lyden ER Hanson CK LeVan TD. Association of dietary fiber on asthma, respiratory symptoms, and inflammation in the adult national health and nutrition examination survey population. Ann Am Thorac Soc. (2020) 17:1062–8. doi: 10.1513/AnnalsATS.201910-776OC, PMID: 32369709

[17] Huffnagle GB. Increase in dietary fiber dampens allergic responses in the lung. Nat Med. (2014) 20:120–1. doi: 10.1038/nm.3472, PMID: 24504401

[18] Hanson C Lyden E Rennard S Mannino DM Rutten EP Hopkins R. The relationship between dietary fiber intake and lung function in the national health and nutrition examination surveys. Ann Am Thorac Soc. (2016) 13:643–50. doi: 10.1513/AnnalsATS.201509-609OC, PMID: 26783997

[19] Szmidt MK Kaluza J Harris HR Linden A Wolk A. Long-term dietary fiber intake and risk of chronic obstructive pulmonary disease: a prospective cohort study of women. Eur J Nutr. (2020) 59:1869–79. doi: 10.1007/s00394-019-02038-w, PMID: 31280344 PMC7351821

[20] Chuang SC Norat T Murphy N Olsen A Tjønneland A Overvad K. Fiber intake and total and cause-specific mortality in the European prospective investigation into cancer and nutrition cohort. Am J Clin Nutr. (2012) 96:164–74. doi: 10.3945/ajcn.111.028415, PMID: 22648726

[21] Park Y Subar AF Hollenbeck A Schatzkin A. Dietary fiber intake and mortality in the NIH-AARP diet and health study. Arch Intern Med. (2011) 171:1061–8. doi: 10.1001/archinternmed.2011.18, PMID: 21321288 PMC3513325

[22] Zipf G Chiappa M Porter KS Ostchega Y Lewis BG Dostal J. National health and nutrition examination survey: plan and operations, 1999-2010. Vital Health Stat 1. (2013):1–37. 25078429

[23] Yang T Yi J He Y Zhang J Li X Ke S. Associations of dietary fats with all-cause mortality and cardiovascular disease mortality among patients with cardiometabolic disease. Nutrients. (2022) 14. doi: 10.3390/nu14173608, PMID: 36079863 PMC9460477

[24] Raper N Perloff B Ingwersen L Steinfeldt L Anand J. An overview of USDA’s dietary intake data system. J Food Compos Anal. (2004) 17:545–55. doi: 10.1016/j.jfca.2004.02.013

[25] Services USDoHaH. Poverty guidelines, research, And measurement. The Available at: http://aspe.hhsgov/POVERTY/index.shtml. (Accessed January 6, 2023).

[26] Qiu Z Chen X Geng T Wan Z Lu Q Li L. Associations of serum carotenoids with risk of cardiovascular mortality among individuals with type 2 diabetes: results from NHANES. Diabetes Care. (2022) 45:1453–61. doi: 10.2337/dc21-2371, PMID: 35503926

[27] Beddhu S Baird BC Zitterkoph J Neilson J Greene T. Physical activity and mortality in chronic kidney disease (NHANES III). Clin J Am Soc Nephrol. (2009) 4:1901–6. doi: 10.2215/CJN.01970309, PMID: 19820134 PMC2798872

[28] Young RP Hopkins RJ Marsland B. The gut-liver-lung Axis. Modulation of the innate immune response and its possible role in chronic obstructive pulmonary disease. Am J Respir Cell Mol Biol. (2016) 54:161–9. doi: 10.1165/rcmb.2015-0250PS, PMID: 26473323

[29] Andrianasolo RM Hercberg S Kesse-Guyot E Druesne-Pecollo N Touvier M Galan P. Association between dietary fibre intake and asthma (symptoms and control): results from the French national e-cohort NutriNet-Santé. Br J Nutr. (2019) 122:1040–51. doi: 10.1017/S0007114519001843, PMID: 31340870

[30] Seyedrezazadeh E Moghaddam MP Ansarin K Asghari Jafarabadi M Sharifi A Sharma S. Dietary factors and risk of chronic obstructive pulmonary disease: a systemic review and meta-analysis. Tanaffos. (2019) 18:294–309. PMID: 32607110 PMC7309892

[31] Fonseca Wald ELA van den Borst B Gosker HR Schols A. Dietary fibre and fatty acids in chronic obstructive pulmonary disease risk and progression: a systematic review. Respirology. (2014) 19:176–84. doi: 10.1111/resp.12229, PMID: 24372903

[32] Kaluza J Harris H Wallin A Linden A Wolk A. Dietary fiber intake and risk of chronic obstructive pulmonary disease: a prospective cohort study of men. Epidemiology. (2018) 29:254–60. doi: 10.1097/EDE.0000000000000750, PMID: 28901975

[33] Afshin A Sur PJ Fay KA Cornaby L Ferrara G Salama JS. Health effects of dietary risks in 195 countries, 1990-2017: a systematic analysis for the global burden of disease study 2017. Lancet. (2019) 393:1958–72. doi: 10.1016/S0140-6736(19)30041-8 30954305 PMC6899507

[34] Kranz S Dodd KW Juan WY Johnson LK Jahns L. Whole grains contribute only a small proportion of dietary fiber to the U.S. Diet Nutr. (2017) 9. doi: 10.3390/nu9020153 PMC5331584 28218657

[35] Xu X Zhang J Zhang Y Qi H Wang P. Associations between dietary fiber intake and mortality from all causes, cardiovascular disease and cancer: a prospective study. J Transl Med. (2022) 20:344. doi: 10.1186/s12967-022-03558-6, PMID: 35918724 PMC9344643

[36] Partula V Deschasaux M Druesne-Pecollo N Latino-Martel P Desmetz E Chazelas E. Associations between consumption of dietary fibers and the risk of cardiovascular diseases, cancers, type 2 diabetes, and mortality in the prospective Nutri net-Sante cohort. Am J Clin Nutr. (2020) 112:195–207. doi: 10.1093/ajcn/nqaa063, PMID: 32369545

[37] Dominguez LJ Bes-Rastrollo M Toledo E Gea A Fresan U Barbagallo M. Dietary fiber intake and mortality in a Mediterranean population: the “Seguimiento Universidad de Navarra” (SUN) project. Eur J Nutr. (2019) 58:3009–22. doi: 10.1007/s00394-018-1846-3, PMID: 30367237

[38] Katagiri R Goto A Sawada N Yamaji T Iwasaki M Noda M. Dietary fiber intake and total and cause-specific mortality: the Japan public health center-based prospective study. Am J Clin Nutr. (2020) 111:1027–35. doi: 10.1093/ajcn/nqaa002, PMID: 31990973

[39] Vaughan A Frazer ZA Hansbro PM Yang IA. COPD and the gut-lung axis: the therapeutic potential of fibre. J Thorac Dis. (2019) 11:S2173–80. doi: 10.21037/jtd.2019.10.40, PMID: 31737344 PMC6831926

[40] Halnes I Baines KJ Berthon BS Mac Donald-Wicks LK Gibson PG Wood LG. Soluble fibre meal challenge reduces airway inflammation and expression of GPR43 and GPR41 in asthma. Nutrients. (2017) 9:57. doi: 10.3390/nu9010057, PMID: 28075383 PMC5295101

[41] Trompette A Gollwitzer ES Yadava K Sichelstiel AK Sprenger N Ngom-Bru C. Gut microbiota metabolism of dietary fiber influences allergic airway disease and hematopoiesis. Nat Med. (2014) 20:159–66. doi: 10.1038/nm.3444, PMID: 24390308

[42] Camilleri M Lyle BJ Madsen KL Sonnenburg J Verbeke K Wu GD. Role for diet in normal gut barrier function: developing guidance within the framework of food-labeling regulations. Am J Physiol Gastrointest Liver Physiol. (2019) 317:G17–g39. doi: 10.1152/ajpgi.00063.2019, PMID: 31125257 PMC6689735

[43] Jang YO Lee SH Choi JJ Kim DH Choi JM Kang MJ. Fecal microbial transplantation and a high fiber diet attenuates emphysema development by suppressing inflammation and apoptosis. Exp Mol Med. (2020) 52:1128–39. doi: 10.1038/s12276-020-0469-y, PMID: 32681029 PMC8080776

[44] Dzierlenga MW Keast DR Longnecker MP. The concentration of several perfluoroalkyl acids in serum appears to be reduced by dietary fiber. Environ Int. (2021) 146:106292. doi: 10.1016/j.envint.2020.106292, PMID: 33395939

[45] Clark ML Butler LM Koh WP Wang R Yuan JM. Dietary fiber intake modifies the association between secondhand smoke exposure and coronary heart disease mortality among Chinese non-smokers in Singapore. Nutrition. (2013) 29:1304–9. doi: 10.1016/j.nut.2013.04.003, PMID: 23911218 PMC9237818

